# Clinical features and prognosis of lung cancer in patients with connective tissue diseases: a retrospective cohort study

**DOI:** 10.3389/fonc.2023.1167213

**Published:** 2023-06-05

**Authors:** Ningning Li, Liwei Gao, Chunmei Bai, Lin Zhao, Yajuan Shao

**Affiliations:** ^1^ Department of Medical Oncology, Peking Union Medical College Hospital, Chinese Academy of Medical Sciences and Peking Union Medical College, Beijing, China; ^2^ Department of Radiation Oncology, China-Japan Friendship Hospital, Beijing, China

**Keywords:** lung cancer, connective tissue diseases, efficacy, survival, therapy

## Abstract

**Background:**

Studies have demonstrated a close association between connective tissue diseases (CTDs) and lung cancer (LC). Evidence supports that poor survival may be associated with the presence of CTDs in patients with LC.

**Methods:**

This retrospective cohort study investigated 29 patients with LC with CTDs, and 116 patients with LC without CTDs were enrolled as case-matched control cohorts. Medical records, therapeutic efficacy of cancer, and outcomes were analyzed.

**Results:**

The median duration from the diagnosis of CTDs to LC was 17 years. The Eastern Cooperative Oncology Group (ECOG) performance score for LC-CTD patients was worse than that for matched non-CTD LC patients. The median progression-free survival (mPFS) and overall survival (mOS) of first-line chemotherapy did not differ between patients with lung adenocarcinoma (AC) with and without CTDs. A significant difference was observed in mPFS [4 months vs. 17 months; hazard ratio (HR), 9.987; *p* = 0.004] and mOS (6 months vs. 35 months; HR, 26.009; *p <* 0.001) of first-line epidermal growth factor receptor tyrosine kinase inhibitor (EGFR-TKI) treatment between patients with AC with and without CTDs. The presence of CTD, sex, ECOG performance status, and tumor-node-metastasis clinical stage were the independent prognostic factors in all patients with non–small cell LC (NSCLC). ECOG performance status was determined to be an independent prognostic factor in patients with LC-CTD. In patients with NSCLC with CTD (n = 26), sex (male) and worse ECOG score were the independent poor prognostic factors.

**Conclusions:**

CTDs were associated with poor survival in patients with LC. The therapeutic efficacy of first-line EGFR-TKI therapy was significantly worse in patients with lung AC with CTDs than in those without CTDs. ECOG performance status was determined as an independent prognostic factor for patients with LC and CTDs.

## 1 Introduction

Connective tissue diseases (CTDs) include systemic lupus erythematosus (SLE), primary Sjögren’s syndrome (pSS), systemic sclerosis (SSc), dermatomyositis/polymyositis (DM/PM), systemic vasculitis (SV), rheumatoid arthritis (RA), and undifferentiated CTD (uCTD) (1). Such disorders are characterized by an autoimmune-mediated systemic inflammatory response that leads to damage to multiple target organs. Evidence suggests that immunological disturbances may promote cancer formation (2). The pathogenic mechanisms are possibly due to chronic exposure to inflammatory mediators, long-term immunosuppression therapy, and dysregulation of the immune system (3). Some cancers may also initiate immune-mediated processes and induce CTDs (4). The relationship between cancer and CTDs is complex.

With the development of industrialization and variations in the atmospheric environment, the disease spectrum in China has changed. Lung cancer (LC) is the leading cause of death in the Chinese population (5). The risk of LC has been reported to be associated with multiple CTDs: SLE, RA, DM/PM, and SSc (6–8). A retrospective study showed that patients with LC with CTDs had a higher incidence of interstitial lung disease (ILD) than those without CTDs. CTD-associated ILD (CTD-ILD) has been proven to be associated with a worse prognosis (9). Patients with pSS with LC were reported to be older than those without cancer, 80% of whom were female non-smokers. Adenocarcinoma (AC) was the most frequently observed cancer pathology in patients with pSS (10). A cohort analysis of 14 SLE cohorts showed that smoking may be the most significant modifiable LC risk factor for SLE (11). A retrospective study compared cancer mortality in a cohort of 122 patients with cancer with CTDs to that in a cohort of 366 patients without CTDs. There were 44 cases of LC with CTDs included in the cohort. The survival of lung cancer was worse in patients with RA or DM/PM than in those without CTDs, whereas SSc was associated with decreased mortality in patients with LC (12).

Although several reports have supported a close association between CTDs and LC, they mostly referred to the association between a certain CTD and LC. Few reports have demonstrated the characteristics of patients with LC with different types of CTDs. Furthermore, accumulating evidence has demonstrated the risk of LC in patients with CTDs and the outcomes for patients with LC with CTDs. Few studies have examined the clinical therapeutic response of these patients, especially the response in patients with LC with different histological types. As cancer prognosis and therapeutic response depend on immune function, we speculated that there may be unique features of treatment efficacy in this CTD-LC population group. Assessing the therapeutic response in this population might contribute to individualized treatments. We aimed to assess the clinical features and compare the treatment response in patients with different pathological diagnoses and the survival of patients with LC with and without CTDs.

## 2 Methods

### 2.1 Study population and design

From January 2014 to December 2017, a total of 5,132 patients with LC were hospitalized at the Peking Union Medical Hospital (PUMCH), 29 of whom had CTDs. We enrolled these 29 patients in the study as the cancer-CTD cohort and retrospectively analyzed their clinical data. As a control cohort (cancer–non-CTD cohort), 116 age-, sex-, cancer type –, and cancer staging–matched LC patients without CTD who were hospitalized at PUMCH during the same period were evaluated. The patients were randomly selected from the medical records to achieve a matching ratio of 1:4 for each cancer-CTD case.

Complete medical data of all patients were obtained. All patients with LC were diagnosed by cytology or histology. The histological types of LC were defined according to the World Health Organization classification in 2004. The cancers were staged on the basis of the American Joint Committee on Cancer (AJCC) seventh staging system. The performance status of patients according to the Eastern Cooperative Oncology Group (ECOG) classification was obtained from medical records (13). Smoking exposure was calculated by the number of packs of cigarettes per day by the number of years for smoking (pack-years) to measure the intensity of smoking. CTDs were diagnosed according to the American College of Rheumatology classification criteria (14–19).

This study enrolled patients with LC treated from 2014 to 2017, during which immune checkpoint inhibitors were not available in China. Thus, owing to drug accessibility and the relative contraindication of CTDs, the application of immunotherapy was relatively rare.

The efficacy of treatment was assessed by objective response rate (ORR), disease control rate (DCR), relapse-free survival (RFS), progression-free survival (PFS), and overall survival (OS), according to response evaluation criteria in solid tumors (RECIST) version 1.1.

The cases and controls were admitted from January 2014 to December 2017 and followed up until March 2021. Medical records were analyzed, including smoking status, body mass index, sequence and duration between the diagnosis of LC and CTDs, metastatic organs, pulmonary embolism (PE), genetic status, treatment and efficacy of LC, CTD status, and patient outcomes.

The study was reviewed and approved by the ethics committee of PUMCH (2017). A written informed consent was obtained from all the participants and guardians of the dead participants in this study.

### 2.2 Statistical analysis

Statistical analyses were performed using IBM SPSS version 22.0 (Chicago, IL, USA). The clinical data are presented as frequency, mean, median, and percentage. Student’s t-tests were used for continuous variables. Nonparametric tests, Pearson chi-squared test, and Fisher’s exact test were used for categorical variables. Analysis of survival and comparisons between groups were performed using the Kaplan–Meier method. Differences in survival were compared using the log-rank test. Follow-up started at the time of cancer diagnosis and was censored at the time of death or on the last day on which survival status was followed up. A two-sided P < 0.05 was considered significant. Forest plots for survival stratified by OS and PFS were performed using GraphPad Prism 8.3.1.

## 3 Results

### 3.1 Demographic and clinical features of patients with LC with CTD

In total, 5,132 patients with LC were hospitalized at PUMCH, of which 29 patients with CTDs and 116 without CTDs were enrolled. During the same period, 3,099 patients with Sjögren’s syndrome, 312 with SSc, 2,622 with SLE, 622 with DM/PM, 547 with uCTD, 761 with SV, and 106 with polymyalgia rheumatica (PMR) were hospitalized in the department of immunology of PUMCH.

We evaluated the clinical characteristics of the 29 and 116 patients with LC with and without CTD, respectively (**Table 1**). Across the LC-CTD cohorts in our study, 75.9% of the patients were female. The median age at onset of LC was 57 years (27–79 years). The median duration from diagnosis of CTD to LC in the 29 patients was 17 years, with the longest interval over 30 years. Ten of these patients developed LC within 1 year after the diagnosis of CTD, even diagnosed simultaneously in some cases. Stage IV disease was more prevalent in patients with LC-CTD. The most common histological type of LC with CTD was AC (19 cases, 73.1%), followed by squamous carcinoma (five cases, 19.2%) and small cell LC (SCLC; three cases, 10.3%).

**Table 1:** Comparisons of the characteristics of patients with LC with or without CTD.

	With CTD (n = 29)	Without CTD (n = 116)	*P -*value
Gender, no. (%)			
Male	7 (24.1)	28 (24.1)	0.605^*^
Female	22 (75.9)	88 (75.9)	
Median age at CTD years (range)	57 (27–79)	N/A	N/A
Median age at cancer years (range)	61 (41–79)	61 (39–78)	0.805^#^
Median years between CTD and LC diagnosis (range)	17 (0–30)	N/A	N/A
TNM staging, no. (%)			
I	3 (10.3)	17 (14.7)	0.398^**^
II	7 (24.1)	19 (16.4)	0.236^*^
IIIA	4 (13.8)	16 (13.8)	0.633^**^
IIIB	1 (3.4)	7 (6.0)	0.455^**^
IIIC	1 (3.4)	2 (1.7)	0.491^**^
IV	13 (44.8)	55 (47.4)	0.484^*^
Histology, no. (%)			
Adenocarcinoma	19 (73.1)	87 (83.7)	0.211^*^
Squamous carcinoma	5 (19.2)	15 (14.4)	0.367^**^
Small cell carcinoma (SCLC)	3 (10.3)	12 (10.3)	0.651^**^
Neurocrine carcinoma (NEC)–non– small cell	2 (7.7)	2 (1.9)	0.179^**^
ECOG PS, no. (%)			
0,1	20 (67.0)	100 (86.2)	0.032^*^
2,3	9 (31.0)	16 (13.8)	
Smoking history, no. (%)			
Yes	7 (24.1)	23 (19.8)	0.388^*^
No	22 (75.9)	93 (80.2)	
Mean number of pack-years of smoking	34.3	46.9	0.280^#^
Metastsis organs, no. (%)			
Intrapulmonary	9 (31.1)	29 (25)	0.329^*^
Pleura	3 (10.3)	29 (25)	0.068^*^
Bone	3 (10.3)	33 (28.4)	0.032^*^
Brain	1 (3.4)	22 (19.0)	0.029^**^
Liver	2 (7.7)	14 (12.1)	0.338^**^
Adrenal gland	2 (7.7)	7 (6.0)	0.570^**^
Pulmonary embolism (PE)	3 (10.3)	9 (7.7)	0.444^**^

* Chi-square test, ** Fisher’s exact test, # Student’s t-test.

Compared to the matched non-CTD LC controls, patients with LC-CTD had a worse ECOG performance score (ECOG ≥ 2: 31.0% vs. 13.8%, *p* = 0.032). Significantly more patients in the non-CTD group were detected with bone metastasis (*p =* 0.032) and brain metastasis (*p* = 0.029) than those in the CTD group. There were no significant differences between the LC-CTD and LC–non-CTD groups with respect to smoking status. On the basis of the pack-year data measured in ever-smokers, smoking exposure was nearly the same between the two groups. Intrapulmonary, pleural, liver, and adrenal gland metastases did not differ between the two groups. There was no significant difference between the LC-CTD and LC–non-CTD groups in terms of age, sex, tumor-node-metastasis (TNM) staging, and PE.

We evaluated the clinical features of the patients in the LC-CTD group according to different pathological types (**Table 2**). Women were predominant in the AC patient subgroup, whereas men were predominant in the SCLC patient subgroup. Patients with SCLC appeared to be younger than those with other cancer histologies. The proportion of patients with an ECOG score ≥2 was significantly higher in the LC-CTD group than in the non-CTD group only in patients with AC (*p* = 0.019). In the AC subgroup, more patients suffered from brain metastasis in non-CTD cases than in CTD cases. The differences in ECOG status and organ involvement between the CTD and non-CTD groups were not significant in the squamous cancer and SCLC subgroups. The morbidity of PE due to high coagulation status did not increase in LC CTD cases compared with that in non-CTD cases in all pathological type subgroups.

**Table 2:** Comparisons of the characteristics of patients with LC-CTD with different pathologic types.

	AC with CTD (n = 19)	AC without CTD (n = 87)	*P-*value	SC with CTD (n = 5)	SC without CTD (n = 15)	*P* value	SCLC with CTD (n = 3)	SCLC without CTD (n = 12)	*P-*value
Gender, no, (%)			_			_			_
Male	3 (15.8)	12 (13.8)		2 (40.0)	8 (53.3)		2 (66.7)	8 (66.7)	
Female	16 (84.2)	75 (86.2)		3 (60.0)	7 (46.7)		1 (33.3)	4 (33.3)	
Median age at cancer-years (range)	62 (41–79)	61 (47–78)	_	61 (50–74)	61 (50–75)	_	55 (41–65)	57 (39–68)	_
ECOG PS ≥ 2, no (%)	4 (21.1)	3 (3.4)	0.019^**^	3 (60.0)	12 (75)	0.366^**^	1 (33.3)	1 (8.3)	0.371^**^
Had smoking history, no. (%)	3 (15.8)	8 (9.2)	0.310^**^	2 (40.0)	7 (46.7)	0.604^**^	2 (66.7)	8 (66.7)	0.736^**^
Mean number of pack-years of smoking	40	16.3	0.852^#^	31.5	58.2	0.513^#^	25	54.4	0.171^#^
PE	3 (15.8)	5 (5.7)	0.152^**^	0 (0)	2(13.3)	0.553^**^	0 (0)	1 (8.3)	0.800^**^
Metastsis organs, no. (%)									
Intrapulmonary	5 (26.3)	24 (27.6)	0.579^*^	2 (40.0)	4 (26.7)	0.483^**^	1 (33.3)	0 (0)	0.200^**^
Pleura	3 (15.8)	20 (23.0)	0.364^**^	0 (0)	3 (20.0)	0.399^**^	0 (0)	5 (41.7)	0.264^**^
Bone	2 (10.5)	21 (24.1)	0.159^**^	1 (20.0)	6 (40.0)	0.406^**^	0 (0)	4 (33.3)	0.363^**^
Brain	0 (0)	17 (19.5)	0.025^**^	1 (20.0)	0 (0)	0.250^**^	0 (0)	4 (33.3)	0.363^**^

AC, adenocarcinoma; SC, squamous carcinoma; SCLC, small cell lung cancer.

* Chi-square test, ** Fisher’s exact test, # Student’s t-test; -, not statistically different.

### 3.2 Clinical characteristics of CTDs in patients with LC

We evaluated the features of CTDs and LC on the basis of the types of CTDs (**Table 3**). Among them, two patients with Sjögren’s syndrome were secondary, suffering from primary SSc and SLE, respectively. Compared with the population of patients with CTDs in our hospital in the same period, they accounted for 0.29% of SS, 1.9% of SSc, 0.15% of SLE, 0.64% of PM, 0.73% of uCTD, 0.39% of SV, and 0.94% of PMR.

**Table 3:** Characteristics of CTDs in 29 patients with lung cancer.

	pSS/sSS (n = 7/n = 2)	SSc (n = 6)	SLE (n = 4)	DM/PM (n = 3/n = 1)	uCTD (n = 4)	SV (n = 3)	PMR (n = 1)
Male, n (%)	0 (0)	0 (0)	1 (25.0)	3 (75.0)	1 (25.0)	2 (66.7)	0 (0)
Female, n (%)	7 (77.8)	6 (100.0)	3 (75.0)	1 (25.0)	3 (75.0)	1 (33.3)	1 (100.0)
Median age at CTD-years (range)	57 (44–72)	39 (27–61)	55 (41–65)	67.5 (55–79)	58 (31–67)	64 (37–64)	64
Median age at cancer-years (range)	58 (50–73)	55 (43–61)	59 (41–65)	67.5 (55–79)	63 (61–74)	64 (41–64)	65
Duration from diagnosis of CTD to LC-years, median (range)	4 (2–6)	10 (8–29)	7 (5–9)	0	10 (7–30)	2.5 (2–3)	1
Diagnosed with LC and CTD simultaneusly, n (%)	3 (33.3)	1 (16.7)	2 (50.0)	4 (100.0)	1 (25.0)	1 (33.3)	0 (0)
CTD-ILD, n (%)	1 (11.1)	5 (83.3)	0 (0)	2 (50.0)	1 (25.0)	2 (66.7)	0 (0)
Had smoking history-n (%)	0 (0)	0 (0)	1 (25.0)	4 (100.0)	1 (25.0)	2 (66.7)	0 (0)
Histology of LC, n (%)							
AC	4 (44.4)	5 (83.3)	3 (75.0)	2 (50.0)	3 (75.0)	1 (33.3)	1 (100.0)
SC	1 (11.1)	1 (16.7)	1 (25.0)	0 (0)	1 (25.0)	1 (33.3)	0 (0)
SCLC	0 (0)	0 (0)	0 (0)	2 (50.0)	0 (0)	1 (33.3)	0 (0)
NEC-NSC	2 (22.2)	0 (0)	0 (0)	0 (0)	0 (0)	0 (0)	0 (0)
CTD relapsed when LC diagnosed, n (%)	0 (0)	2 (33.3)	0 (0)	0 (0)	1 (25.0)	2 (66.7)	0 (0)
Treatment of CTDs, n (%)							
Corticosteroids	3 (33.3)	4 (66.7)	1 (25.0)	4 (100.0)	2 (50.0)	2 (66.7)	1(100.0)
Cyclophosphamide	0 (0)	1 (16.7)	0 (0)	0 (0)	0 (0)	0 (0)	0 (0)
Methotrexate	0 (0)	2 (33.3)	1 (25.0)	0 (0)	0 (0)	2 (66.7)	0 (0)
Others*	2 (22.2)	2 (33.3)	3 (75.0)	0 (0)	1 (25.0)	0 (0)	0 (0)

pSS, primary Sjögren’s syndrome; sSS, secondary Sjögren’s syndrome; SSc, systemic sclerosis; SLE, systemic lupus erythematosus; DM/PM, dermatomyositis polymyositis; uCTD, undifferentiated connective tissue disease; SV, systemic vasculitis; PMR, polymyagia rheumatica; CTD-ILD, connective tissue disease-associated interstitial lung disease; AC, adenocarcinoma; SC, squamous carcinoma; SCLC, small cell lung cancer; NEC-NSC, non–small cell neurocrine carcinoma.

* Others: pristimerin, azathioprine, hydroxychloroquine, mycophenolate mofetil, and cyclosporin.

There was a female predominance in the pSS/sSS, SSc, SLE, uCTD, and PMR subgroups, whereas males patients were dominant in the DM/PM (75%) and SV (66.7%) subgroups. The median age at onset of CTD in the SSc subgroup was 39 (range, 27–61) years, which was much younger than that in the other CTD subgroups. In the SSc and uCTD subgroups, the duration from diagnosis of CTDs to LC was long as 7–30 years, mostly more than 10 years. However, most patients in the pSS/sSS, SLE, DM/PM, SV, and PMR subgroups were identified as having LC within 5 years of CTD diagnosis. All patients in the DM/PM subgroup and half of those in the SLE subgroup were confirmed to have LC and CTDs simultaneously.

AC was predominant in most CTD subgroups, except for DM/PM and SV cases. SCLC accounted for 50% and 33.3% of the cases in the DM/PM and SV subgroups, respectively, which was significantly higher than that in the other subgroups. CTD-ILD was mostly observed in patients with SSc (83.3%), followed by SV (66.7%) and DM/PM (50%). All patients in the DM/PM subgroup were ever-smokers. Four of the 29 patients had CTD relapse due to LC onset. Most patients received corticosteroid treatment, whereas some received disease-modifying antirheumatic drugs (DEMARDs).

### 3.3 Evaluation of the therapeutic efficacy of patients with LC with and without CTD

#### 3.3.1 Efficacy of the adjuvant therapy in patients with AC with and without CTD

During the observation period, three of the 19 patients with AC with CTD and 16 of the 87 patients with AC without CTD were considered to have stage I disease. All stage I patients in both groups underwent lobectomy. Four patients with stage IB disease in the non-CTD group received adjuvant systemic chemotherapy with a cisplatin-based regimen. All stage I patients remained disease-free during the follow-up period.

Four patients with AC with CTDs had stage II disease. Two of the patients received adjuvant chemotherapy with a cisplatin-based doublet after lobectomy, whereas the other two patients with epidermal growth factor receptor (EGFR) mutation received adjuvant tyrosine kinase inhibitor (TKI) treatment. RFS was 2, 14, and 16 months, respectively, in three patients. RFS has not yet been reached in one patient. Three patients died, and the OS was 12, 16, and 20 months, respectively. Among AC without CTD cases, 17 were confirmed as stage II disease after surgery. Sixteen patients received a cisplatin-based doublet regimen, and one was administered anaplastic lymphoma kinase (ALK) inhibitors as adjuvant therapy. Disease progression was determined in three of the 17 patients with RFS of 26, 26, and 27 months, respectively. None of the 17 patients died during the follow-up period and appeared to have better efficacy and survival than those in the CTD group.

In the patients with AC with CTD, three were considered to have stage IIIA disease. Two patients received radical surgery, and one patient received only supportive care instead of surgery due to poor respiratory function and skin status because of SSc. After surgery, one patient received TKI as adjuvant therapy and has remained disease-free for more than 29 months. One patient received pemetrexed plus cisplatin chemotherapy with RFS of 8 months and OS of 21 months. The patients who received supportive care died from cancer 8 months after diagnosis. Among AC patient–matched non-CTD cases, 12 patients were confirmed to have stage IIIA disease after radical surgery. Four patients with EGFR mutation received EGFR-TKI, and eight patients underwent doublet chemotherapy after surgery. The 12 patients have survived for 20–62 months.

#### 3.3.2 Efficacy of the treatment in patients with advanced or metastatic AC with and without CTD

Sufficient clinical evaluation helped to confirm the initial diagnosis of nine patients with stage IV AC in the LC-CTD group. Three patients received supportive care only because of CTDs. One patient had muscle weakness because of DM, which became the main reason for the choice of palliative care. The other two patients received supportive care because of severe PE (SV) and thrombocytopenia (uCTD), respectively. Four patients with AC in the LC-CTD group received combined chemotherapy as initial treatment. All four patients received platinum-based doublet chemotherapy for four to six cycles. Maintenance with pemetrexed was administered to two patients for three to 15 cycles until progression. One of the four patients withdrew from chemotherapy because of severe thrombocytopenia. The ORR of first-line chemotherapy was 25%, whereas the DCR was 75%. The incidence of grade 3 hematological toxicity was almost 50% among those receiving chemotherapy. Treatment with EGFR-TKI as a single agent was indicated for the initial treatment of two patients with identified driver mutations. Both patients showed rapid disease progression at the fourth month of treatment, and the OS was 6 and 9 months, respectively. Among the nine patients with stage IV AC in the LC-CTD group, only one patient received second-line treatment with serious chemotherapy-related adverse events (PFS, 1.5 months; OS 9, months). Because of the relative contraindication of CTDs, the patients in the LC-CTD group were not treated with immunotherapy.

There were 41 patients with stage IV AC in the matched non-CTD group. All patients received systemic treatment. Immune checkpoint inhibitors were not approved in China in 2014–2017; thus, most of the patients in the control group were not prescribed immunotherapy. Nineteen patients (three of 19 were EGFR mutation-positive) received a platinum-based regimen as initial treatment. Five of these patients received a combination of chemotherapy with EGFR monoclonal antibodies or vascular endothelial growth factor monoclonal antibodies, whereas one of the 19 patients received chemotherapy combined with pembrolizumab with unknown programmed death-ligand 1 (PD-L1) expression. The ORR of first-line chemotherapy was 10.5%, and the DCR was 78.9%. Among the 41 patients with advanced AC in the non-CTD group, 21 patients were treated with TKI as first-line treatment, including 20 with EGFR inhibitors and one with ALK inhibitor. The tumors contained an EGFR exon 19 deletion (n = 9) or an exon 21 (n = 10, L858R mutation) substitution mutation, including the rare exon 18 G719x mutation (n-1). Twenty patients with EGFR mutation were administered gefitinib (n = 10), icotinib hydrochloride (n = 5), erlotinib (n = 3), afatinib (n = 1), or osimertinib (n = 1). One patient with ALK fusion oncogene was treated with ALK-TKI crizotinib. The ORR of first-line TKI was 28.6%, and DCR was 90.5%. One patient with tumor PD-L1 Tumor cell Proportion Score (TPS) expression >1%, without activating EGFR or ALK alterations, received a programmed death-1 (PD-1) blocker and had stable disease (SD). The PFS with PD-1 blocker of this patient was 7 months, whereas OS was not reached (>24 months).

**Figure 1:**
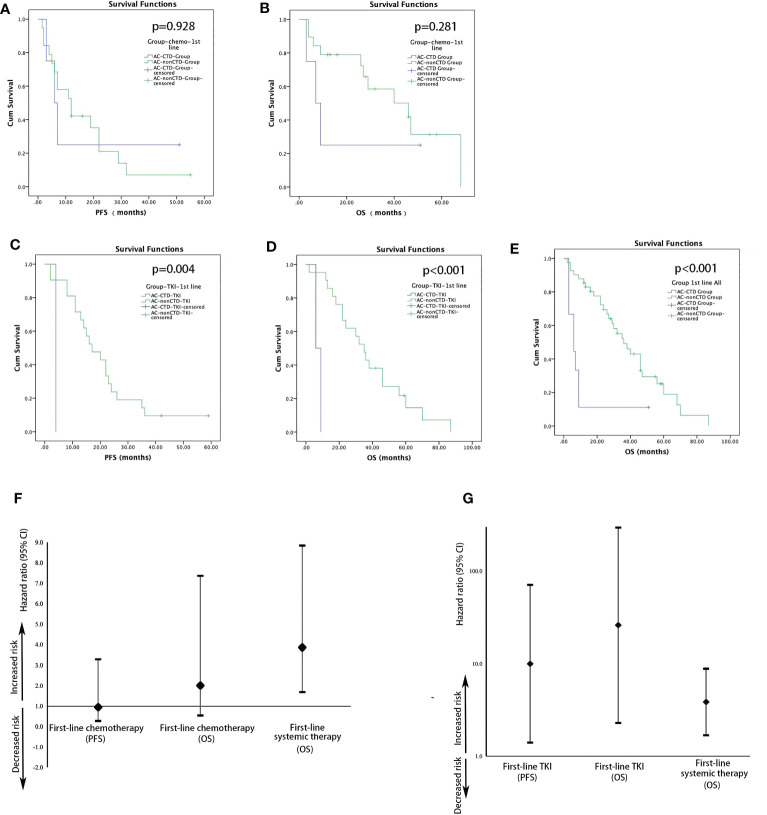
Kaplan–Meier estimates of the probability of PFS and OS in patients with AC with first-line treatment. **(A)** Comparison of PFS in patients with AC with or without CTD receiving chemotherapy (12 vs. 6 months; HR, 1.057; 95% CI, 0.304–3.678; p = 0.928). **(B)** Comparison of OS in patients with AC with or without CTD receiving chemotherapy (46 vs. 7 months; HR, 2.005; 95% CI, 0.546–7.361; p = 0.281). **(C)** Comparison of PFS in patients with AC with or without CTD receiving TKI (17 vs. 4 months; HR, 9.987; 95% CI, 1.406–70.945; p = 0.004). **(D)** Comparison of OS in patients with AC with or without CTD receiving TKI (35 vs. 6 months; HR, 26.009; 95% CI, 2.293–295.077; p < 0.001). **(E)** Comparison of OS in patients with AC with or without CTD receiving first-line treatment (p < 0.001). Forest plots for survival stratified by PFS and OS for patients with AC with first-line chemotherapy **(F)** or TKI **(G)**.

During the follow-up period, five patients with advanced AC with CTD and 36 patients with advanced AC without CTD showed disease progression after initial treatment. Five patients with AC in the CTD group and 20 patients in the non-CTD group died during this time interval. There were no significant differences in the median PFS (mPFS) of first-line chemotherapy between the patients with AC with (n = 4) or without CTD (n = 19) [12 vs. 6 months; hazard ratio (HR), 0.946; 95% confidence interval (CI), 0.272–3.291; *p* = 0.928; **Figures 1A, F**]. First-line chemotherapy also resulted in no significant differences in OS between the CTD and non-CTD group in AC (46 vs. 7 months; HR, 2.005; 95% CI, 0.546–7.361; *p* = 0.281; **Figures 1B, F**). A significant difference was observed in the mPFS and median OS (mOS) of first-line TKI treatment between the two groups. mPFS of TKI treatment in the AC-CTD group was 4.0 months (n = 2), whereas mPFS was 17.0 months in the AC–non-CTD group (n = 21) (HR, 9.987; 95% CI, 1.406–70.945; *p* = 0.004; **Figures 1C, G**). mOS in the AC-CTD and AC–non-CTD groups was 6.0 and 35.0 months, respectively (HR, 26.009; 95% CI, 2.293–295.077; *p* < 0.001; **Figures 1D, G**). Regardless of the therapy regimen, the mOS of all patients with advanced lung AC with initial treatment was significantly different between the two groups. Patients without CTD had better OS than patients with CTD (36 vs. 6 months; HR, 3.861; 95% CI, 1.686–8.842; *p* < 0.001; **Figures 1E-G**).

#### 3.3.3 Efficacy of the treatment in patients with SC with and without CTD

Patients with SC in CTD (n = 3) and non-CTD (n = 5) groups with stage I–IIIA underwent radical surgery. No significant difference was observed in DFS after adjuvant therapy.

In the CTD group, two patients with stage IIIB-IV SC were treated with first-line platinum-based chemotherapy. The mPFS was 2 months, and the mOS was 2.5 months. There were 10 patients with SC in the non-CTD group (n = 10). Six patients received first-line chemotherapy (gemcitabine + cisplatin, vinorelbine + cisplatin, and etoposide + cisplatin). They all exhibited a SD in efficacy evaluation. The mPFS was 6 months, and the mOS was 10 months. One patient was treated with immunotherapy with partial response efficacy. The PFS was 8 months, and the OS was 36 months. Three non-CTD patients received the best supportive care. Their OS was 3, 14, and 20 months, respectively.

#### 3.3.4 Efficacy of the treatment in patients with SCLC with and without CTD

One patient with localized stage (LS)–SCLC in the CTD group was diagnosed after radical surgery at the early stage. DFS was not reached (>55 months) after adjuvant chemotherapy (etopside and cisplatin). Six patients with LS-SCLC in the non-CTD group were treated with first-line chemotherapy (etoposide + platinum). The mPFS was 13 months, and the mOS was 38.5 months.

Of the two patients with extensive stage (ES)–SCLC in the CTD group, one received chemotherapy with a PFS of 5 months and OS of 5.5 months, and the other received the best supportive care with an OS of 3 months. Six patients with ES-SCLC in the non-CTD group received first-line chemotherapy with an mPFS of 11 months and an mOS of 24 months.

### 3.4 Analysis of the survival and prognostic factors for LC in patients with and without CTD

**Figure 2:**
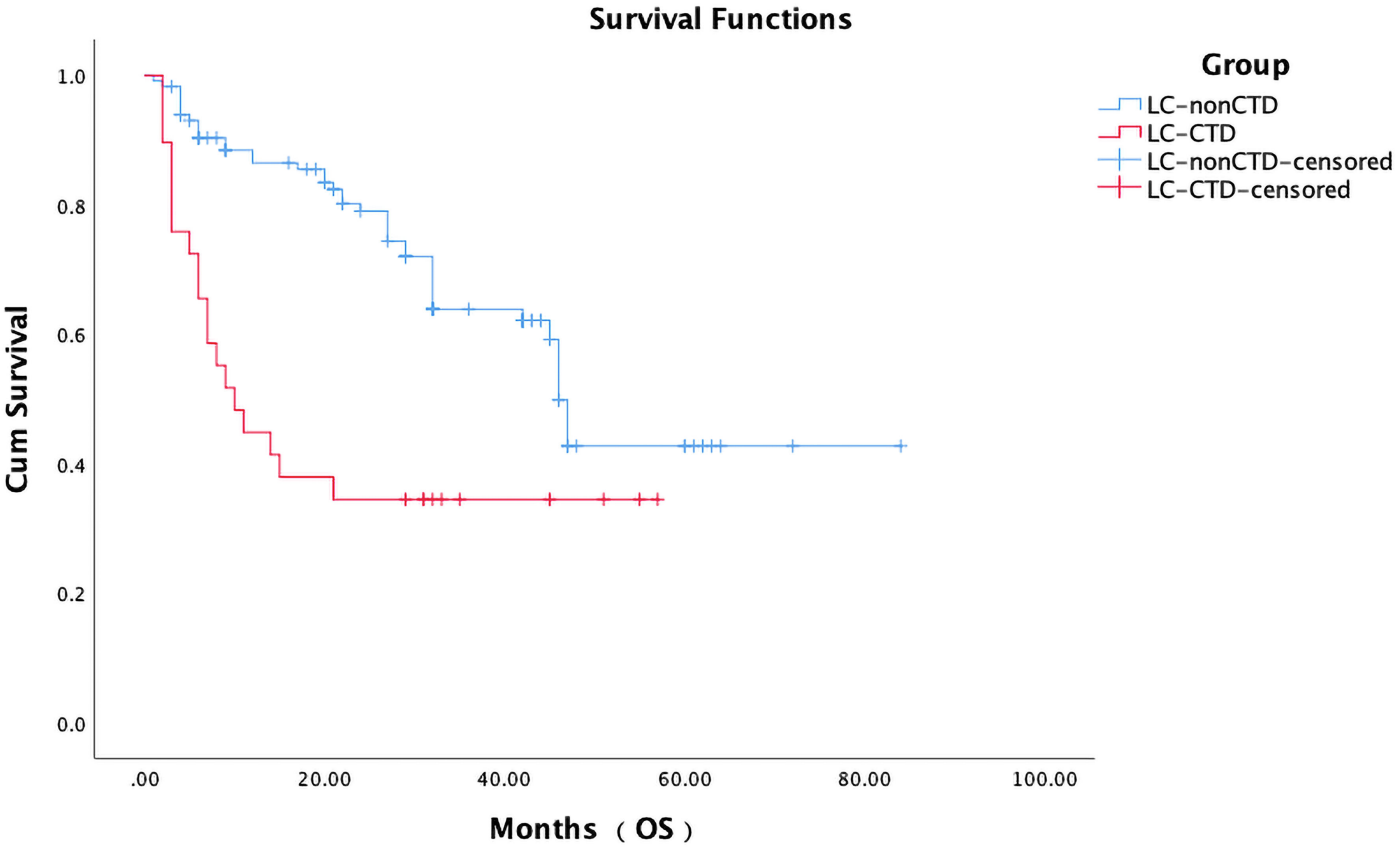
The median OS of all patients with advanced lung adenocarcinoma with initial treatment. Patients without CTD had better OS than patients with CTD (36 vs. 6 months; HR, 3.861; 95% CI, 1.686–8.842; p < 0.001).

During the follow-up period, 19 patients with LC with CTD (65.5%) and 41 patients with LC without CTD (35.3%) died. There were significant differences in the mOS between the LC-CTD and LC non-CTD cohorts (46 months vs. 10 months; HR, 2.685; 95% CI, 1.554–4.638; p = 0.000; **Figure 2**). We further investigated the potential prognostic factors in all patients with LC (n = 145). In the univariate analysis, sex (male), worse ECOG performance score, late TNM clinical stage, pathological type SCLC, smoking history, PE, and the presence of a CTD were significantly associated with poor prognosis (**Table 4**). On the basis of the univariate analysis results, variables of sex, ECOG score, TNM clinical stage, pathological type, smoking history, PE, and the presence of CTD were included in a multivariate Cox regression analysis. The presence of CTD (HR, 4.452; 95% CI, 2.489– 7.963; p = 0.000), sex (HR, 2.187; 95% CI, 1.284–3.726; p = 0.004), and TNM clinical stage (HR, 8.754; 95% CI, 3.844–19.934; p = 0.000) were determined as the independent prognostic factors in patients with LC (**Table 4**). In patients with non–small cell LC (NSCLC) (n = 130), multivariate Cox regression analysis demonstrated that CTD, sex, ECOG performance status, and clinical stage were the independent prognostic factors (**Supplementary Table 1**).

**Table 4:** Univariate and multivariate analyses for death of all patients with LC (n = 145).

Variables	Univariable analysis		Multivariable analysis	
	HR (95% CI)	P -value	HR (95% CI)	P- value
Age				
(>61 vs. ≤61years)	–	0.811	–	–
Gender				
(male vs. female)	3.865 (2.321–6.435)	0.000	2.187 (1.284–3.726)	0.004
ECOG				
(2,3 vs. 0,1)	5.107 (2.832–9.210)	0.000	–	0.136
TNM clinical stage				
(IIIb,IV vs. I–IIIa)	8.934 (4.053–19.694)	0.000	8.754 (3.844–19.934)	0.000
Pathology				
(SCLC vs. NSCLC)	2.024 (1.052–3.895)	0.045	–	0.849
Smoking history				
(yes vs. no)	3.443 (2.054–5.770)	0.000	–	0.385
CTD				
With CTD vs. without CTD	2.685 (1.554–4.638)	0.000	4.452 (2.489–7.963)	0.000
Pulmonary embolism				
(yes vs. no)	2.895 (1.412–5.936)	0.004	–	0.085

SCLC, small cell lung cancer; NSCLC, non–small cell lung cancer; -, not applicable.

We also investigated the prognostic factors in patients with LC with CTD (n = 29) (**Table 5**). Although ECOG performance status, clinical stage, and smoking history all demonstrated significant prognostic potential in univariate analysis, in further multivariate analysis, only ECOG performance status was determined as an independent prognostic factor (HR, 5.578; 95% CI, 2.155–14.43; p = 0.000). In patients with NSCLC with CTD (n = 26), sex (male) and a worse ECOG score were the independent poor prognostic factors (**Supplementary Table 2**).

**Table 5:** Univariate and multivariate analyses for death of patients with LC with CTD (n = 29).

Variables	Univariable analysis		Multivariable analysis	
	HR (95% CI)	P- value	HR (95% CI)	P- value
Age				
(≤61 vs. >61 years)	–	0.139	–	0.310
Gender				
(male vs. female)	–	0.056	–	0.207
ECOG				
(2,3 vs. 0,1)	5.578 (2.155–14.438)	0.000	5.578 (2.155–14.438)	0.000
TNM clinical stage				
(IIIb,IV vs. I–IIIa)	5.240 (1.832–14.984)	0.002	–	0.055
Pathology				
(SCLC vs. NSCLC)	–	0.859	–	–
Smoking history				
(yes vs. no)	2.499 (0.973–6.419)	0.057	–	0.581
Pulmonary embolism				
(yes vs. no)	–	0.859	–	–
ILD				
(yes vs. no)	–	0.190	–	–
Duration between the diagnosis of CTD and LC				
Within 1 year vs. more than 1 year	–	0.488	–	–

SCLC, small cell lung cancer; NSCLC, non–small cell lung cancer; ILD, interstitial lung disease.

### 3.5 Cause of death in patients with and without CTD

During the observation period, 19 of the 29 patients with LC-CTD and 41 of 116 patients with LC–non-CTD died.

Thirteen patients in the LC-CTD cohort died of cancer, and six died from CTD. Three of the six patients with CTD died from CTD-ILD (3 of 11, 27.3%). These three patients were diagnosed with SSc, SV, and DM, respectively. One patient with SLE died of renal failure caused by lupus nephritis. One patient with PM died of respiratory failure caused by respiratory muscle weakness. One patient died from PE, ST elevation myocardial infarction (STEMI), and cerebral infarction due to SV.

All 41 patients in the LC–non-CTD cohort died of LC or cancer-associated complications.

## 4 Discussion

Some studies have analyzed the risk and mortality of LC in patients with CTDs. The highest standardized incidence ratios (SIRs) and standardized mortality ratios were shown in discoid lupus erythematosus (4.71 and 4.80), polymyositis/dermatomyositis (4.20 and 4.17), SLE (2.47 and 2.69), and SSc (2.19 and 1.98) (20). This study confirmed an increased risk of developing LC. For patients with LC and CTDs, our study revealed that female patients were more frequently seen, which may be due to the high incidence of CTDs in female patients. However, male dominance was observed in the DM/PM and SV subgroups. A meta-analysis investigated the risk of malignancy among patients with DM and PM (21), which showed that the risk of malignancy is higher among male than female patients with DM, which is consistent with our reports.

The present study demonstrated that the duration from diagnosis of CTDs to LC varied from 0 to 30 years, including some patients diagnosed within a year. Consistent with previous reports, one study (22) of LCs arising in CTDs (SSc, RA, and SLE) suggested that, on average, LCs tend to arise late in the course of CTDs (an average of 13.9; range, 0–30 years after CTD diagnosis). This suggests a tendency toward delayed diagnosis of cancers occurring in CTDs. Most patients in the DM/PM and SLE groups were diagnosed simultaneously, which is similar to the results of the present study. Similar findings showed that the risk of cancer among patients with DM was highest in the first year after diagnosis and then steadily decreased from years 2 to 5 (21). The highest cancer SIR in the first year after SLE diagnosis was also evaluated by some reports (23). DM/PM and SLE may also manifest in a paraneoplastic fashion. A report of patients with juvenile SSc and lung AC suggested that SSc may represent a paraneoplastic syndrome related to LC (24). In one study, myositis autoantigen expression was revealed to be increased in cancer-associated myositis, suggesting that cancer may be an antigen source initiating an immune response (25).

The pathological types of LC in patients with CTDs were AC, squamous carcinoma, and small cell carcinoma, which are also common in the general population of patients with LC. In addition, the proportion of each histological type was 73.1%, 19.2%, and 10.3%, respectively, which is consistent with the data reported in the general population (26). We observed that all histologies were affected in both patients with DM/PM and SV, with a higher risk of small cell carcinoma. An autoimmune paraneoplastic response may contribute to this status. Previous studies have shown a similar correlation between SLE and SCLC (20); however, the mechanism is not clear. CTD-ILDs were frequently seen in patients with SSc, SV, and DM/PM in our study. A retrospective analysis suggested that a higher prevalence of ILD was observed in patients with LC with CTD than in those without CTD (52% vs. 14%) (9), whereas the incidence of ILD in the CTD cohort was 37.9% (11 of 29) in the present study. The presence of CTD-ILD is regarded as an independent poor prognostic factor in patients with LC with CTD (9). Our results suggest that ILD is not associated with a worse outcome for LC in patients with CTD.

An aim of this study was to estimate the influence of CTD on LC therapeutic efficacy. Patients with CTD had a worse therapeutic response than LC-matched controls. We found that the efficacy of first-line EGFR-TKI in CTD patients with AC was much worse than that in non-CTD patients. The poor response may be due to the ECOG performance status and poor drug tolerance during treatment. At present, among the treatment options for LC, targeted therapy is the most important means to improve the survival time for patients with driving gene mutations. The incidence of EGFR mutation in the Asian population is approximately 62% (27). Studies have shown that EGFR-TKI is the first-line treatment for people with EGFR mutation, with an mPFS of 18.9 months (28) and a total mOS of 38.6 months (29), which is consistent with the control group results in our study. According to the present study, the mPFS of EGFR-TKI in patients with CTDs was only 4 months, significantly worse than that of the control group (17 months). Similarly, the mOS of patients with CTD was 6 months, which was much worse than that of the control group (35 months). This poses a greater challenge for clinicians to choose a treatment strategy in this population. The reason and mechanisms for the poor response to EGFR-TKI in patients with CTDs have never been reported. This needs to be verified in further investigation.

Immune checkpoint inhibitors have been approved and widely applied since 2018; therefore, only one patient was treated with immunotherapy in the current study. Currently, immunotherapy is the first-line treatment for patients with LC without sensitive genetic mutations. For patients with pre-existing CTDs, there are limited data on the safety and efficacy of checkpoint inhibitor immunotherapy (30). Clinical trials evaluating immunotherapy exclude patients with CTDs because of concerns about the deterioration of underlying autoimmune diseases or immune-related adverse events (irAEs) (31). Observational studies have suggested that some patients with CTDs can safely receive immunotherapy (30, 32–36). However, compared with patients without CTDs, those with CTDs may have a higher risk of developing specific irAEs or discontinuing immunotherapy because of irAEs (30). These patients may also be at a higher risk of developing underlying CTD exacerbation (31, 32, 34, 36). A prospective observational cohort study included 4,367 patients with advanced melanoma, of whom 415 (10%) had CTDs (30). A total of 228 (55%) of the patients with CTDs received immunotherapy. The incidence of irAEs >3 was similar to that of patients with melanoma without CTDs. Among patients treated with PD-1 inhibitors, compared with patients without CTDs, patients with CTDs were more likely to discontinue immunotherapy because of irAEs (17% vs. 9%). However, data on the acute exacerbation of preexisting CTDs have not been reported. For these patients, clinicians should carefully discuss the advantages and disadvantages before starting immunotherapy and evaluate several clinical factors. For patients with life-threatening CTDs, immunotherapy should be used with extreme caution or avoided (37). For patients with LC, data on the application of immunotherapy in patients with CTDs are limited. We hope that this study can provide some background information for carrying out further investigation in this patient population.

Although the present study did not show a significant difference in chemotherapeutic efficacy between patients with LC with CTDs and the patients in the control cohort, there was still a descending trend in the comparison of PFS between groups. We deduce that the impacts of CTDs on patients with LC may include poor function reserve of organs, poor performance status, and immunologic hematologic involvement, which lead to difficulties in chemotherapy tolerance. This will also affect the prognosis of patients with cancer.

The treatment strategy and outcome determine cancer survival. Some studies have investigated the prognostic factors in cancer-CTD populations. One study suggested that the presence of ILD caused by CTDs is an independent poor prognostic factor in patients with LC with CTD. Patients with LC and CTD-ILD failed to receive standard therapy because of poor respiratory function. For instance, some patients with CTD-ILD were treated with chemotherapy alone instead of chemoradiotherapy because of ILD. Severe radiation pneumonitis occurred in these patients after radiotherapy (9). In the present study, ILD was not a significant prognostic factor for CTD patients with LC in the Cox analysis, although three of the 11 patients with ILD died of ILD exacerbation, which seemed high. Patients with CTDs are well known to present with ILD, which is a common form of organ dysfunction in CTD. Clinicians should focus more on this disease. More research should be carried out to confirm if the existence of CTD-ILD is a poor prognostic factor.

Nearly half of the patients with LC and CTDs in our sample died from CTDs. Their higher mortality compared with the mortality in patients with common LC implies the poor prognosis of these patients. Consistent with the present study, a retrospective cohort study (12) demonstrated that the prognosis of lung or breast cancer was worse in patients with RA or DM/PM than in those without CTDs. However, results from another study demonstrated that CTDs have no large influence on OS, except that some CTDs appear to impair survival in small cell carcinoma (20). In addition to the prognostic significance of CTDs, we also found that male sex was a poor independent prognostic factor in all patients with LC and was also significant in patients with NSCLC with CTD. Previous studies have suggested that SLE that occurs in men tends to have worse outcomes (38); however, data on the association between male sex and prognosis of other types of CTDs is limited. Within the group of patients with CTDs, the ECOG performance status score showed prognostic significance in the multivariate Cox regression analysis. Performance status is an important index for predicting the prognosis of cancer, especially for patients with CTDs, which commonly affect multiple organs.

This study had several limitations. First, the sample size of the cohort was relatively small owing to the rarity of the disease. Furthermore, the diagnostic procedures of CTDs were performed in the Department of Immunology by different clinicians; therefore, the duration of definite diagnosis and treatment strategy were not strictly consistent. Third, the current study included patients from 2014 to 2017, during which the principle treatment was different from that used today, especially in the aspect of the application of immunotherapy and targeted therapy. In addition, because of the small sample size, we did not compare and evaluate the differences in efficacy and prognosis between different types of CTDs. The use of steroids and DMARDs prescribed to manage CTDs might have affected the treatment choices, tolerance of chemotherapy, and the outcome of standard systemic therapy. Finally, the patients included in the current study were all hospitalized, which may have led to the worse baseline condition of the enrolled population. These limitations should be addressed in future larger prospective studies.

## 5 Conclusions

The prognosis of patients with CTD-LC was worse than that of patients with LC only. Furthermore, the therapeutic response to first-line EGFR-TKIs was much worse in patients with lung AC with CTDs than in those without CTDs. ECOG performance status was determined as an independent prognostic factor for patients with LC and CTDs. These results imply that clinicians should pay special attention to management strategies in this LC-CTD population. To the best of our knowledge, this is the first study to investigate the efficacy of CTDs in LC. Further studies are needed to establish rational therapeutic strategies to overcome the treatment limitations and improve the survival of patients with both LC and CTD.

## Data availability statement

The raw data supporting the conclusions of this article will be made available by the authors, without undue reservation.

## Ethics statement

The study was reviewed and approved by the ethics committee of Peking Union Medical Hospital. The patients/participants provided their written informed consent to participate in this study.

## Author contributions

NL and CB designed the study. NL and LG analyzed the data and wrote the manuscript. CB and LZ contributed to the data interpretation and the revision of the manuscript. NL, LZ, and YS performed the clinical data collection. All authors contributed to the article and approved the submitted version.

## Glossary

CTDs: connective tissue diseases
SLE: systemic lupus erythematosus
pSS: primary Sjögren’s syndrome
SSc: systemic sclerosis
DM/PM: dermatomyositis/polymyositis
SV: systemic vasculitis
RA: rheumatoid arthritis
UCTD: undifferentiated CTD
LC: lung cancer
ILD: interstitial lung disease
PUMCH: Peking Union Medical Hospital
AJCC: American Joint Committee on Cancer
ECOG: Eastern Cooperative Oncology Group
ORR: objective response rate
DCR: disease control rate
RFS: relapse-free survival
PFS: progression free survival
OS: overall survival
RECIST: response evaluation criteria in solid tumors
PMR: polymyalgia rheumatica
SCLC: small cell lung cancer
PE: pulmonary embolism
DEMARDs: disease-modifying antirheumatic drugs
AC: adenocarcinoma
EGFR: epidermal growth factor receptor
TKI: tyrosine kinase inhibitor
ALK: anaplastic lymphoma kinase
PD-L1: programmed death-ligand 1
PD-1: programmed death 1
SD: stable disease
HR: hazard ratio
CI: confidence interval
SC: squamous carcinoma
LS: localized stage
ES: extensive stage
NSCLC: non–small cell lung cancer
STEMI: ST elevation myocardial Infarction
SIRs: standardized incidence ratios
SMRs: standardized mortality ratio.
