# Supplementary material for: Clinical features and prognosis of lung cancer in patients with connective tissue diseases: a retrospective cohort study
Source: Front Oncol. 2023 Jun 5;13:1167213. doi: 10.3389/fonc.2023.1167213 (PMC10277622; doi:10.3389/fonc.2023.1167213)
Supplement: Supplementary file 1 [file Table_1.docx]

Supplementary Material

Clinical features and prognosis of lung cancer in patients with connective tissue diseases: A retrospective cohort study

Ningning Li^1^, Liwei Gao^2^，Chunmei Bai*^1^, Lin Zhao^1^, Yajuan Shao^1^

*** Correspondence:** Chunmei Bai: baichunmei@pumch.cn

# Supplementary Tables

**Supplementary Table 1** Univariate and multivariate analyses for death of all NSCLC patients (n=130).

| Variables | Univariable analysis | | Multivariable analysis | |
| --- | --- | --- | --- | --- |
|  | HR(95% CI) | P value | HR(95% CI) | P value |
| Age  (≤61 vs. >61 years) | - | 0.812 | - | - |
| Gender  (Male vs. Female) | 4.454（2.508-7.909） | 0.000 | 2.224（1.156-4.279） | 0.017 |
| ECOG  (2,3 vs. 0,1) | 6.558 (3.443-12.490） | 0.000 | 2.082（1.038-4.177） | 0.039 |
| TNM clinical stage  （Ⅲb，Ⅳ vs. I-Ⅲa ） | 7.814（3.505-17.418） | 0.000 | 5.780（2.436-13.713） | 0.000 |
| Smoking history  (Yes vs. No) | 4.417（2.413-8.088） | 0.000 | - | 0.959 |
| CTD  With CTD vs. Without CTD | 3.141（1.738-5.679） | 0.000 | 4.316（2.254-8.265） | 0.000 |
| Pulmonary embolism  (Yes vs. No) | 3.139（1.456-6.767） | 0.004 | - | 0.183 |

NSCLC：non-small cell lung cancer

**Supplementary Table 2** Univariate and multivariate analyses for death of NSCLC patients with CTD (n=26).

| Variables | Univariable analysis | | Multivariable analysis | |
| --- | --- | --- | --- | --- |
|  | HR(95% CI) | P value | HR(95% CI) | P value |
| Age  (≤61 vs. >61 years) | - | 0.177 | - | - |
| Gender  (Male vs. Female) | 5.444（1.679-17.649） | 0.005 | 5.087（1.459-17.741） | 0.011 |
| ECOG  (2,3 vs. 0,1) | 6.128（2.237-16.789） | 0.000 | 6.087（2.113-17.534） | 0.001 |
| TNM clinical stage  （Ⅲb，Ⅳ vs. I-Ⅲa ） | 4.476（1.537-13.039） | 0.006 | - | 1.172 |
| Smoking history  (Yes vs. No) | 4.074（1.457-11.391） | 0.007 | - | 0.995 |
| Pulmonary embolism  (Yes vs. No) | - | 0.820 | - | - |
| ILD  （Yes vs. No） | - | 0.540 | - | - |
| Duration between the diagnosis  of CTD and LC  Within one year  More than one year | - | 0.286 | - | - |

NSCLC：non-small cell lung cancer
